# Prevalence and factors associated with severe anaemia amongst under-five children hospitalized at Bugando Medical Centre, Mwanza, Tanzania

**DOI:** 10.1186/s12878-015-0033-5

**Published:** 2015-10-12

**Authors:** Rehema H. Simbauranga, Erasmus Kamugisha, Adolfine Hokororo, Benson R. Kidenya, Julie Makani

**Affiliations:** Department of Paediatrics and Child health, Catholic University of Health and Allied Sciences, Box 1464, Mwanza, Tanzania; Department of Biochemistry and Molecular Biology, Catholic University of Health and Allied Sciences, Box 1464, Mwanza, Tanzania; Department of Paediatrics and Child health, Bugando Medical Centre, Box 1370, Mwanza, Tanzania; Department of Haematology and Blood Transfusion, School of Medicine, Muhimbili University of Health and Allied Sciences (MUHAS), Box 65001, Dar- Es- Salaam, Tanzania

**Keywords:** Severe anaemia, Under-five, Mwanza, Tanzania

## Abstract

**Background:**

Anaemia is a major public health problem in developing countries, contributing significantly to morbidity and mortality amongst children under-five years of age. About 43 % of under-fives are anaemic worldwide, and two-thirds reside in sub-Saharan Africa. Even where blood transfusion is available for treatment there is still a significant case fatality rate ranging between 6 and 18 %. This study aimed to determine the prevalence and morphological types of anaemia, as well as factors associated with severe anaemia in under-five children admitted at Bugando Medical Centre (BMC).

**Methods:**

This was a hospital-based, cross-sectional study conducted between November 2012 and February 2013. Selected laboratory investigations were done on children admitted to BMC. Anaemia was defined using WHO criteria.

**Results:**

A total of 448 under-five children were recruited into the study. The overall prevalence of anaemia was 77.2 % (346/448) with mild, moderate and severe anaemia being 16.5, 33 and 27.7 % respectively. Microcytic hypochromic anaemia was detected in 37.5 % of the children with anaemia. Of 239 children with moderate and severe anaemia, 22.6 % (54/239) had iron deficiency anaemia based on serum ferritin level less than12 μg/ml. The factors associated with severe anaemia included unemployment of the parent, malaria parasitaemia and presence of sickle haemoglobin.

**Conclusion:**

The prevalence of anaemia among under-five children admitted at BMC was high. Iron deficiency anaemia was the most common type. Factors associated with severe anaemia were unemployment among caretakers, malaria parasitaemia and presence of sickle haemoglobin.

## Background

Anaemia is a major public health problem in developing countries [1]. The global estimate of childhood anaemia indicates that 293.1 million (approximately 43 %) of children under five years are anaemic worldwide and 28.5 % of these children are residing in sub-Saharan Africa (sSA) [1]. It is considered a major public health problem reaching 67 % prevalence, equivalent to 83.5 million children in sSA [1]. Anaemia is one of the most common causes of mortality of children admitted to hospitals in Sub-Saharan Africa. Anaemia can be managed without blood transfusion but even where blood transfusion is available there is still a significant case fatality rate of 6–18 % [2]. In East Africa approximately 75 % of under-five children have anaemia [3] with the prevalence’s ranging between 44 and 76 % [2–4]. The risk factors for anaemia vary in different settings; they include having intestinal parasites, malaria, HIV infection, nutritional deficiencies and habit of taking tea with meals, haematological malignancies and chronic diseases like sickle cell disease (SCD) [5–8]. Anaemia in childhood may also result from factors such as poor socioeconomic status and maternal health status including presence of iron deficiency anaemia [2]. The hospital records from Bugando Medical Centre (BMC) indicate that many children are admitted with anaemia, and severe anaemia is stated to be amongst the top causes of admission and mortality in the paediatric wards. Unfortunately, the true burden remains unknown as no studies have been conducted in this setting. This study aimed to determine the prevalence of mild, moderate and severe anaemia, morphological types of anaemia as well as factors associated with severe anaemia among children under-five years admitted at BMC paediatric wards.

## Methods

### Study site and subjects

This was a hospital-based, cross sectional, prospective study conducted at BMC, in Mwanza. BMC is one of the four referral hospitals in Tanzania, located in the northwestern part of Tanzania, it admits 10,000 children aged 6 to 59 months annually.

### Patient recruitment

Recruitment was done serially after obtaining an informed consent. All children aged 6–59 months who were admitted at BMC and their guardian or parents consented to participate in the study were included. Children with active haemorrhage, bleeding disorders, history of blood transfusion and/or surgery within two months prior to admission were excluded. All children were managed according to BMC paediatric treatment guidelines.

### History taking and physical examination

Information regarding patients age, sex, residence, birth weight, natal history of prematurity, breast feeding practices, age at introduction of other types of food, types of complementary food in the first year of life, habit of taking tea and 24 h dietary recall were obtained from parents/guardians. Additional information that was taken included family history of SCD, history of chronic illness, blood transfusion and being on medication. Caretaker occupation, information about family size and education level of the parents were also obtained. Recruited patients were assessed for conjunctival and palmar pallor. A thorough physical examination of these children was performed. Nutritional evaluation was done according to WHO reference standard for malnutrition (WHO technical report series, 1995) for under-five years of age using Z-score (see Table 1) and participants were classified into mild, moderate and severe malnutrition.

**Table 1:** Operational Definitions

Term	Definition
Anaemia	Was defined by using WHO classification of anaemia as haemoglobin level of less than 11 g/dl [32].
Mild anaemia	Was defined by haemoglobin level of 10 to 10.9 g/dl.
Moderate anaemia	Was defined by haemoglobin level of 7 to 9.9 g/dl.
Severe anaemia	Was defined by haemoglobin level of less than 7 g/dl.
Iron deficiency	Was defined as serum ferritin concentration <12 μg/l.
Iron deficiency anaemia	Was defined as concurrent anaemia and iron deficiency.
Serum ferritin and iron deficiency	Was corrected for infection using C-reactive protein therefore Serum ferritin level was considered low when C-reactive protein was negative and serum ferritin level of <12 μg/ml or when C-reactive protein was positive and serum ferritin level of <30 μg/ml [33].
Z-score(SD-score)	Was equal to (observed value – median value of reference population)/standard deviation value of reference population
Mild malnutrition	Defined as per WHO guidelines was considered when weight for height/length is between median and -1SD.
Moderate malnutrition	Defined as per WHO guidelines was considered when weight for height/length is between -2SD and -3SD (between 70–80 %) with no oedema
Severe malnutrition	Was defined as the presence of oedema of both feet, or severe wasting or both (weight for height/length < 70 % or ≤ −3SD).
Morphological type of anaemia	Was defined as the erythrocyte shape, size and haemoglobinisation as seen on light microscopic examination of the blood film
Normochromic	Was a descriptive term applied to a red blood cell with a normal amount of colour within the red blood cell.
Hypochromic	Was a descriptive term applied to a red blood cell with a decreased concentration of haemoglobin where the cells have an expanded central zone of pallor greater than one-third of the diameter of the cell.
Peasant	A traditional class of farmers who owns small farms, in other words a none employee or business person

### Laboratory investigations

Haemoglobin estimation was done by Haemo_control EKF diagnostic machine (EKF diagnostics, Germany). All participants with haemoglobin less than 11 g/dl by haemocue had a complete blood count using an automated haematology analyzer CELL DYN 3700 (Abbott Laboratories, USA). Sickling test was done to detect the presence of haemoglobin S using the sodium metasulphite test. Haemoglobin electrophoresis was done at Muhimbili National Hospital (MNH) laboratory in Dar es salaam, Tanzania to identify haemoglobin genotypes in all children with positive sickling test as well as those with negative sickling test but with symptoms and signs, and peripheral smear results suggestive of SCD. Peripheral smear (using Leishman stain, Ranbaxy, India) was done to look at morphological characteristics of anaemia. Serum ferritin (Ferritin ELISA, Genway Biotech, USA) and C - reactive protein (Direct Latex test, Veda Lab, France) were done in all children with moderate and severe anaemia. Blood smear for malaria parasites was done using Giemsa stain (Ranbaxy, India) and the number of asexual parasite/200WBC was counted. To ensure the quality of the blood slides for malaria parasites and peripheral blood smears, two readings were done by two different experienced laboratory technologists. A laboratory scientist was consulted as a third reader when discordance was noted and Quality control was done by experienced laboratory scientist. HIV testing was done according to the national algorithm. For children below 18 months, DNA PCR for HIV was done in children with positive rapid test, and for those with negative rapid test, but born to HIV positive mothers [9]. For children with HIV positive test, CD4 count (FACS Calibur, BD Germany) was done to detect the severity of immune suppression. All operational definitions used in this study were as defined in Table 1.

### Data management and Statistical analysis

Data were double entered using Microsoft Excel 2007, cleaned and analyzed using STATA version 11 (StataCorp, Texas). Categorical variables were reported as proportion and were compared using either Pearson chi squared test or Fischer’s exact test for small numbers. Continuous data were described as means (standard deviation) or medians (interquartile range) depending on the distribution of data. To determine factors associated with severe anaemia univariate and multivariate logistic regression analyses were done. Factors with a *p*-value of ≤ 0.1 on univariate logistic regression were subjected to multivariate logistic regression analysis. Crude and adjusted odds ratios were calculated to quantify the strength of association between severe anaemia and risk factors. The 95 % confidence interval was determined and risk factors with a *p*-value of less than 0.05 were considered significant.

### Ethical approval

Ethical clearance was obtained from the CUHAS-BMC ethics committee. The protocol and importance of the study was explained to the participants’ parents or guardians before recruitment into the study, followed by a signed informed consent by a parent or guardian. Those who refused to participate in the study were given service equal to that of all the other children. Treatment was done according to the BMC paediatric department protocol.

### Morphological classification of anaemia

This was done by morphological examination of the peripheral blood film. Seven different morphological types of anaemia were observed; microcytic hypochromic, normocytic normochromic, mixed hypochromic, macrocytic normochromic, mixed normochromic, macrocytic hypochromic and microcytic normochromic anaemia [10].

*Mixed (anisocytosis) means RBCs containing microcytic and macrocytic cells.

### Haemoglobin categories for sickle status

Haemoglobin categories were as described below:

Presence of two normal haemoglobin **(AA)**

Presence of homozygous sickle haemoglobin **(SS).** Also known as sickle cell anaemia.

Presence of one normal haemoglobin and the other is sickle haemoglobin **(AS).** Also known as sickle cell trait.

Presence of fetal haemoglobin **(HbF)**

### Indications for testing for SCD by Haemoglobin electrophoresis

The indications for doing haemoglobin electrophoresis were positive sickling test or a peripheral smear suggesting the presence of SCD or first line family with SCD. Also, the presence of physical features suggestive of SCD like bossing of the skull, malocclusion of teeth and gingival hypertrophy were taken into consideration.

## Results

### Prevalence of anaemia

For the period from November 2012 to February 2013, 1,050 children were admitted to BMC paediatric wards. Of these, 602 were out of age limit and 448 children aged 6 to 59 months were eligible for determination of prevalence of anaemia. Anaemia was found in 346 (77.2 %) children. The prevalence of mild, moderate and severe anaemia were 16.5 % (74/448), 33 % (148/448) and 27.7 % (124/448) respectively.

### Baseline characteristics of children with anaemia

**Fig. 1:**
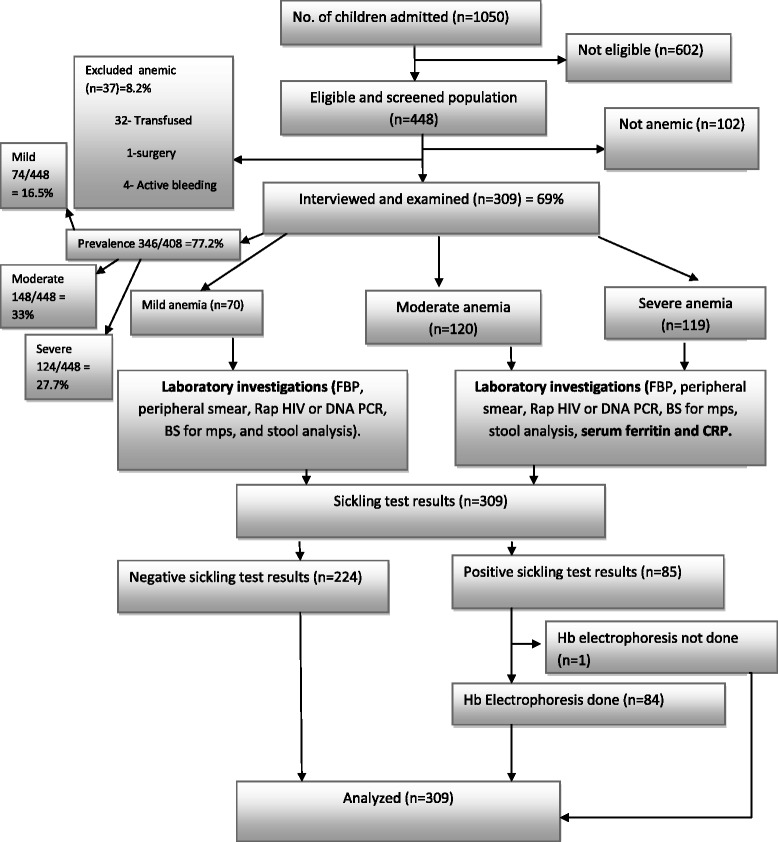
Recruitment flow chart

To analyze for morphological types of anaemia and factors associated with severe anaemia, 37 children were excluded from 346 children, due to the following reasons; 32 children had blood transfusion prior to admission, 1 child underwent surgery and 4 children had active bleeding. Therefore 309 children were interviewed and examined (Fig. 1). Of 309 children 57.3 % (177/309) were male and 67.3 % (208/309) were below 2 years. The mean age was 23.2 months [IQR 11–33]. Eighty eight percent (272/309) of the children were from Mwanza region. Majority of caretakers were peasants 48.9 % (151/309) and 65.7 % (203/309) had at least primary education. Seventy nine percent (244/309) were weaned earlier than 6 months and 38.8 % (120/309) were severely malnourished. Median haemoglobin was 7.7 g/dl [IQR 5.7–9.7] and according to severity of anaemia median haemoglobin levels for mild, moderate and severe anaemia were 10.4 g/dl [IQR 10.1–10.8], 8.3 g/dl [IQR 7.6–9.1] and 5.2 g/dl [IQR 4.4–6.2] respectively (Table 2).

**Table 2:** Baseline characteristics of 309 children <5 years of age with anaemia

Variable	Frequency	Percentage (%)
Sex		
Male	177	57.3
Female	132	42.7
Age		
≤ 2 years	208	67.3
> 2 years	101	32.7
Caretaker education		
No formal education	67	21.6
Primary	203	65.7
Secondary	32	10.4
College/University	7	2.3
Caretaker occupation		
Housewife	52	16.8
Employed by institution	29	9.4
Small scale business	77	24.9
Peasant	151	48.9
Nutritional status		
Normal	77	24.9
Mild Malnutrition	74	24.0
Moderate Malnutrition	38	12.3
Severe Malnutrition	120	38.8
Birth Order		
≤ 4 babies	209	67.6
> 4 babies	100	32.4
Delivery status		
Preterm	7	2.3
Term	302	97.7
Birth weight(kg)		
< 2.5	32	10.4
≥ 2.5	277	89.6
Weaning period		
≤ 6 month	244	79.0
> 6 months	65	21.0

### Morphological types of anaemia

**Fig. 2:**
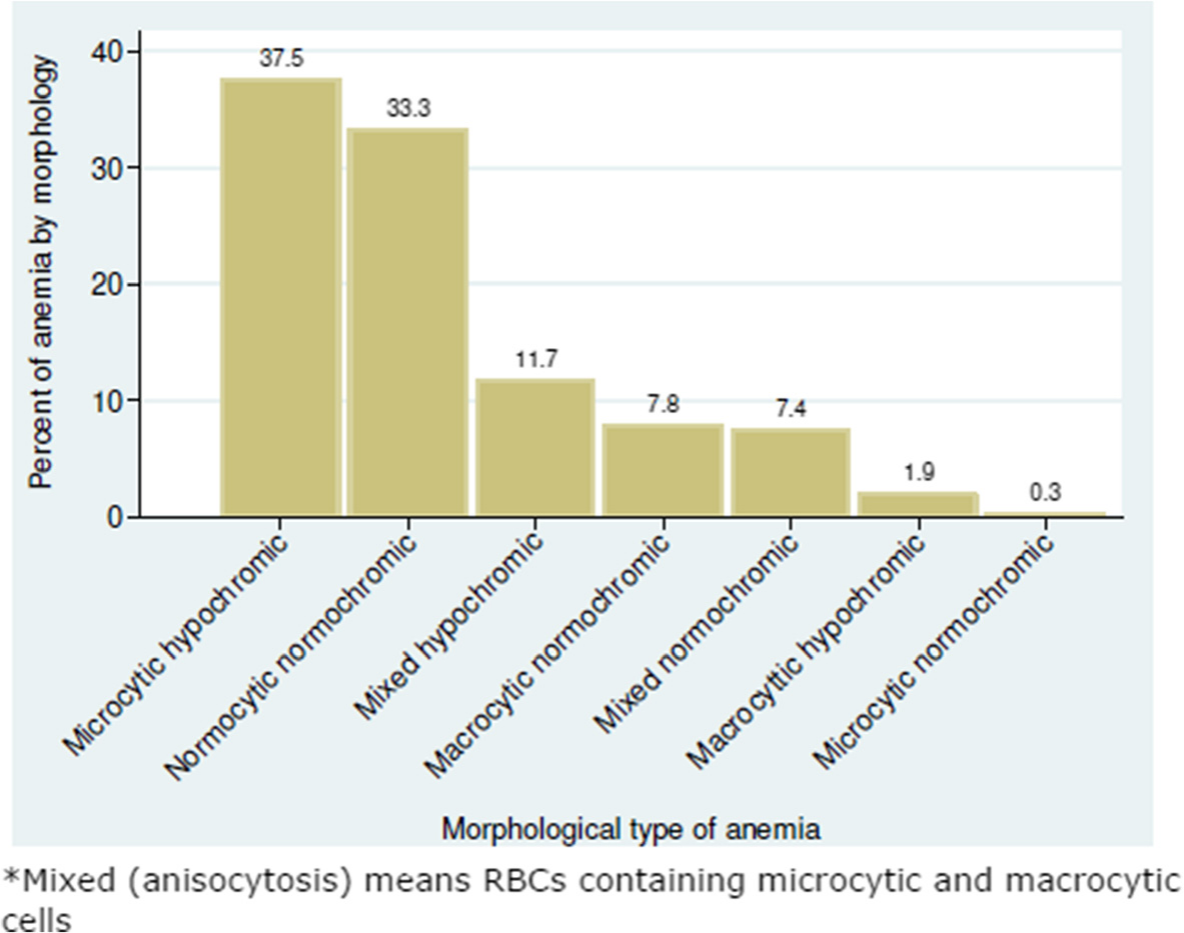
Morphological types of anemia

The commonest type of anaemia was microcytic hypochromic anaemia occurring in 37.5 % (116/309) of children, followed by 33.3 % (103/309) with normocytic normochromic anaemia. One child (0.33 %) had microcytic normochromic anaemia (Fig. 2).

### Factors associated with severe anaemia

On univariate analysis, factors associated with severe anaemia were child’s age >2 years [OR = 1.9; 95 % CI = 1.1 - 3.0; *p*-value = 0.012] and habit of taking tea with meals [2.1 (1.3–3.4); 0.002]. Low level of caretaker’s education [2.4 (1.1–5.3); 0.031], unemployment of caretakers [2.1 (1.3–3.5); 0.004] and malaria parasitaemia [4.0 (2.2–7.3); 0.001] were also associated with severe anaemia. On multivariate analysis, the factors associated with severe anaemia were unemployment [2.2 (1.2–4.0); 0.007], malaria parasitaemia [4.0 (2.1–7.8); <0.001] and presence of haemoglobin S [2.0 (1.1–3.5); 0.018] (Table 2). Low birth weight and HIV were not significantly associated with severe anaemia. There was no significant association between severe anaemia and nutritional status i.e. wasting (mild malnutrition, moderate malnutrition and severe malnutrition) [Table 3].

**Table 3:** Factors associated with severe anaemia

Risk factor	Anaemia		Unadjusted		Adjusted	
	Severe N (%)	Mild to Moderate n (%)	OR [95 % CI]	*P*-value	OR [95 % CI]	*P*-value
Child age						
> 2 years	70 (33.7)	138 (66.4)	1			
≤ 2 years	49 (48.5)	52 (51.5)	1.9 [1.1–3.0]	0.012	1.1 [0.6–2.1]	0.827
Sex						
Female	46 (34.9)	86 (65.1)	1			
Male	73 (41.2)	104 (58.8)	1.3 [0.8–2.1]	0.254	-	-
Birth weight						
≥ 2.5 kg	104 (37.6)	173 (62.4)	1			
< 2.5 kg	15 (46.9)	17 (53.1)	1.5 [0.7–3.1]	0.307	-	-
Tea taking habit						
No	45 (29.6)	107 (70.4)	1			
Yes	74 (47.1)	83 (52.9)	2.1 [1.3–3.4]	0.002	1.7 [0.9–3.2]	0.110
Malaria parasitaemia						
No	81 (32.3)	170 (67.7)	1			
Yes	38 (65.5)	20 (34.5)	4.0 [2.2–7.3]	<0.001	4.0 [2.1–7.8]	<0.001
Presence of haemoglobin S						
No	79 (35.3)	145 (64.7)	1			
Yes	40 (47.1)	45 (52.9)	1.6 [1.0–2.7]	0.058	2.0 [1.1–3.5]	0.018
Nutrit. status						
Normal	35 (45.5)	42 (54.5)	1			
Mild	33 (44.6)	41 (55.4)	1.0 [0.5–1.8]	0.915	0.6 [0.3–1.3]	0.209
moderate	12 (31.6)	26 (68.4)	0.6 [0.2–1.3]	0.157	0.4 [0.1–0.9]	0.028
severe	39 (32.5)	81 (67.5)	0.6 [0.3–1.0]	0.068	0.5 [0.3–1.0]	0.053
C- educ. level						
Sec. or above	9 (23.1)	30 (76.9)	1			
Primary	85 (41.9)	118 (58.1)	2.4 [1.1–5.3]	0.031	2.0 [0.8–4.8]	0.121
Non-formal	25 (37.3)	42 (62.7)	2.0 [0.8–4.9]	0.133	1.3 [0.5–3.5]	0.669
C- occupation						
Employed	29 (27.4)	77 (72.6)	1			
Unemployed	90 (44.3)	113 (55.7)	2.1 [1.3–3.5]	0.004	2.2 [1.2–4.0]	0.007
C- HIV status						
No	112 (38.6)	178 (61.4)	1			
Yes	7 (36.8)	12 (63.2)	0.9 [0.4–2.4]	0.877	-	-

Serum ferritin was done on children with moderate and severe anaemia. Of the 239 children, 22.6 % (54/239) had low serum ferritin levels; the proportion of those with low serum ferritin levels was higher in the group with moderate anaemia 53.7 % (29/54) compared to 46.3 % (25/54) with severe anaemia. This was not statistically significant (*p*-value = 0.559). Among 309 patients of the study population, 27.5 % (85/309) fulfilled the criteria for haemoglobin electrophoresis. One of the caretakers declined electrophoresis testing in one patient; therefore 27.2 % (84/309) samples were processed. Of these 40.5 % (34/84) were SS, 36.9 % (31/84) were AS and 19 % (16/84) were AA. SS and AS were significantly associated with severe anaemia (*p*-value = 0.012).

### Correlation between Serum ferritin and morphological types of anaemia

Among the children with moderate and severe anaemia, 41 % (98/239) had microcytic hypochromic anaemia. In correlating morphological types of anaemia and serum ferritin levels, 90.9 % (50/55) of patients with low serum ferritin levels had microcytic hypochromic anaemia (Table 4).

**Table 4:** Correlation between serum ferritin and Morphological types of anaemia

Morphological type of anaemia	Serum ferritin levels		
	Low	Normal	High
Microcytic hypochromic anaemia	50 (90.9)	24 (31.2)	24 (22.4)
Normocytic normochromic anaemia	2 (3.6)	29 (37.6)	29 (27.1)
Mixed hypochromic anaemia	3 (5.5)	8 (10.4)	19 (17.8)
Macrocytic normochromic anaemia	0 (0.0)	5 (6.5)	18 (16.8)
Mixed normochromic anaemia	0 (0.0)	8 (10.4)	13 (12.2)
Macrocytic hypochromic anaemia	0 (0.0)	3 (3.9)	3 (2.8)
Microcytic normochromic anaemia	0 (0.0)	0 (0.0)	1 (0.9)

## Discussion

In this study the prevalence of anaemia was 77.2 % with mild, moderate and severe anaemia being 16.5, 33 and 27.7 % respectively. The overall prevalence of anaemia in this study was similar to that reported from other studies in Tanzania, Kenya and South Africa where the prevalence of anaemia was between 71 and 79 % [11–13]. The prevalence of severe anaemia was high in this study. Muoneke et al. [6] reported a lower prevalence of severe anaemia of 9.7 % in Nigeria. The higher prevalence of severe anaemia in this study may be due to higher prevalence of malaria, nutritional deficiencies and SCD. Sickle cell disease and malaria were significantly associated with severe anaemia in this study. Muoneke et al. [6] found a significant association between SCD and SCT with severe anaemia in Nigeria. Children with SCD are susceptible to infections which may result in rapid fall of haemoglobin level. Mechanisms which contributes to severe anaemia in SCD are chronic haemolysis or hyper-haemolytic crisis, folate or iron deficiency resulting from increased utilization of folate and enhanced urinary loss of iron, depression of erythropoiesis (aplastic crisis) and sequestration crisis [14]. Similar to our findings, several studies have also reported that malaria is an important cause of severe anaemia [8, 11, 15, 16]. It contributes to anaemia through red blood cell lysis, organ sequestration and destruction of erythrocytes, phagocytosis of uninfected and infected red blood cells and dyserythropoiesis [17].

The morphological classification of anaemia in the screened patients showed that microcytic hypochromic anaemia was the most predominant type, occurring in 37.5 % of this population. It is assumed that iron depletion is the main factor responsible for the high proportion of microcytic-hypochromic anaemia. This finding is supported by the correlation between serum ferritin and morphological types of anaemia where 90.9 % of patients with low serum ferritin levels had microcytic hypochromic anaemia. Nutrition is the likely cause of iron deficiency. Seventy nine percent of the study children started weaning at the age of 6 months or younger. Majority had supplementary feeding with rice based foods that lack both iron and protein, which is likely to contribute to iron deficiency during infancy and childhood. Iron deficiency anaemia has been found to impair production of IL 2 and IL 6 and as a result it increases susceptibility to infections [18]. It is important to bear in mind that anaemia of chronic infection and inflammation, haemoglobinopathies such as thalassemia as well as sideroblastic anaemia may also present with microcytic hypochromic erythrocyte morphology [19].

Unemployment was found to be significantly associated with severe anaemia in our study, similar to other reports [20, 21]. Unemployment and low level of education might lead to poor socio-economic status. This suggests that better socio- demographic conditions increases access to better nutrition and health care and as a result lower the risk of anaemia. In this study, we also found that lower level of education was associated with increased risk of anaemia. Kahigwa et al. reported that anaemic children had caretakers who did not complete primary education [16]. The relationship between education and anaemia may be due to the capacity of caretakers to grasp the knowledge needed for adequate healthcare and nutrition for children.

Merhav et al. 1985, Wilson et al. 1999 and Zaida et al. 2006 reported that habit of taking tea with meals was significantly associated with anaemia [22–24]. It has been reported that tea contributes to anaemia due to iron deficiency because it contains polyphenol which inhibit iron absorption. Polyphenol in the intestines binds to iron and form non transportable complex which will be excreted in faeces [25]. In this study univariate analysis showed significant association between habit of tea taking with meals and severe anaemia but in multivariate analysis this factor was not significant statistically. Villamor et al., 2000 found that children older than 2 years of age were at a higher risk of anaemia than those below 2 years [8]. In this study we found a significant association between age above 2 years and severe anaemia in univariate analysis but not in multivariate analysis. Direct association between malaria and age is likely to be the cause in this study. Prevalence of malaria increased with age, 26.7 % in children older than 24 months versus 14.9 % in children less or equal to 24 months (*p*-value 0.012). Also, increased prevalence of anaemia in children above 2 years in this study may be an indicator of persistent anaemia after treatment of malaria due to prolonged immune response and associated activation of macrophages [26]. Most of the children had more than one attack of malaria.

In this study low birth weight was not associated with severe anaemia and this may be due to the small number (10.4 %) of the children with low birth weight. A similar finding was reported by Kahigwa et al., 2002 [16] in a case control study. Malnutrition is among the causes of anaemia as reported by other researchers [16, 27]. In this study, there was no significant association between nutritional status and severe anaemia, similar to the findings by Ughasoro et al. 2011 [28]. Nutrition status was evaluated using weight for height; the reason for this contrary association was not clear in our study population. This might be due to reductive adaptation occurring in malnourished children whereby the reduction in red cell mass liberates free iron. The free iron will then be converted to ferritin for storage and this may reduce the risk of developing severe anaemia.

HIV infection is known to be among the causes of anaemia in children in the developing world [8, 29–31]. However, in this study, there was no significant association between HIV and anaemia. The reasons could be a small number of children who were HIV positive and the regular attendance to HIV care and treatment centre.

### Limitations of the study

As a referral hospital, it is likely that the patients were highly selected, with the more severe forms of anaemia being seen in this setting. There was limitation in diagnostic facilities to determine causes of anaemia. This included unavailable folate levels, B12 levels, thalassemia, G6PD deficiency and bone marrow biopsy for those with possible bone marrow failure.

## Conclusions

Prevalence of anaemia in children admitted at BMC is high, with significant proportion having mild and moderate anaemia. Microcytic, hypochromic and normocytic normochromic pattern were the common morphological types of anaemia. Factors associated with severe anaemia included unemployment among caretakers, malaria parasitaemia, and presence of sickle haemoglobin. We recommend routine screening for anaemia in all admissions and treatment with haematinics to the patients who will be diagnosed. Supplementation of infants and treatment of iron deficiency is recommended. Since children whose parents or caretakers are unemployed have a high risk of having anaemia, further studies are needed to identify factors to address this issue. Establishing the SCD status is important in this setting.

## Abbreviations

BMC: Bugando Medical Centre
CRF: Case record form
MNH: Muhimbili National Hospital
CUHAS: Catholic University of Health and Allied Sciences
IQR: Interquatile range
CTC: Care and Treatment Centre
SCD: Sickle cell disease
SCT: Sickle Cell trait
